# A Microfluidic Device for Continuous Sensing of Systemic Acute Toxicants in Drinking Water

**DOI:** 10.3390/ijerph10126748

**Published:** 2013-12-03

**Authors:** Xinyan Zhao, Tao Dong

**Affiliations:** Department of Micro and Nano Systems Technology (IMST), Faculty of Technology and Maritime Sciences (TekMar), Vestfold University College (HiVE), Tønsberg N3103, Norway; E-Mail: td@hive.no

**Keywords:** microfluidic device, *Vibrio fischeri*, cell toxicity, bioluminescence, counter-flow micromixer

## Abstract

A bioluminescent-cell-based microfluidic device for sensing toxicants in drinking water was designed and fabricated. The system employed *Vibrio fischeri* cells as broad-spectrum sensors to monitor potential systemic cell toxicants in water, such as heavy metal ions and phenol. Specifically, the chip was designed for continuous detection. The chip design included two counter-flow micromixers, a T-junction droplet generator and six spiral microchannels. The cell suspension and water sample were introduced into the micromixers and dispersed into droplets in the air flow. This guaranteed sufficient oxygen supply for the cell sensors. Copper (Cu^2+^), zinc (Zn^2+^), potassium dichromate and 3,5-dichlorophenol were selected as typical toxicants to validate the sensing system. Preliminary tests verified that the system was an effective screening tool for acute toxicants although it could not recognize or quantify specific toxicants. A distinct non-linear relationship was observed between the zinc ion concentration and the Relative Luminescence Units (RLU) obtained during testing. Thus, the concentration of simple toxic chemicals in water can be roughly estimated by this system. The proposed device shows great promise for an early warning system for water safety.

## 1. Introduction

Drinking water is one of the most important resources in everyday life. With industrial development and urban expansion, contamination accidents sometimes threaten the safety of the drinking water system [1]. The detection of possible toxicants in drinking water is an essential task for urban water suppliers. Frequent measurements of chemicals in drinking water can reduce the possibility of accidents related to pollutants or contaminants; however, the safety of water can also be influenced by local incidents. On-line early warning systems are widely employed at most stages of the urban water cycle, including intake, treatment and distribution, whereas household drinking water monitoring is not available at present [2,3]. Thus, there are relatively few local systems in place to guarantee real-time public health protection for drinking water. The need for monitoring toxicants in drinking water in home has led to the idea of low-cost devices that can continuously monitor systemic toxicant chemicals, such as heavy metal ions and phenol, thus providing early warning signals. Currently, systems for rapid and continuous analysis of toxicant contaminants are limited to laboratories, although analytical methods for a broad range of organic and inorganic chemicals are available [4]. Many commercially available technologies used for the detection of routine water quality continue to provide the most reliable means of detecting anomalies within the drinking water system, but most of these technologies require complex instrumentation and protocols that are not amenable to household operation [2,5,6]. Moreover, traditional chemical tests often allow highly accurate determination of only a set of targets, whereas unknown toxicants in the same sample might go undetected. Living-organism-based detection is a rapid and authoritative method to evaluate the total toxicity of aqueous samples, instead of only measuring the concentrations of a set of chemical constituents [6,7]. For example, certain fish can serve as living models for environmental toxicity assessment. When the fish in a river begin to show anomalies, there is an indication of toxicants in the water. The Toxprotect™ fish monitor (bbe moldaenke GmbH, Schwentinental, Germany) has been developed to detect toxins in water by analyzing the swimming activity of up to 20 fish across an array of 80 photoelectric light diode barriers in real time [2]. However, animal models have a high resistance to many toxicants and are the subject of ethical issues. Moreover, they have high maintenance costs. Cell-based toxicity tests provide an alternative to animal experiments. These systems can monitor a broad range of chemicals, including some unknown chemicals [8].

Model mammalian or human cells display similar physiological responses to the cells in the human body; however, it can be difficult to maintain the viability of mammalian cells and there are huge challenges associated with continuous detection of toxicants in mammalian systems [9]. Compared with mammalian cells, bioluminescent bacteria have a higher growth rate and less costly to propagate. Bacteria are also easy to detect and have shorter response times to various agents [10]. Thus, bioluminescent bacteria represent promising sensors for use in continuous-sensing devices against toxicants. Additional advantage of using bioluminescent bacterium sensors is generally attributed to the minimal ethical standards associated with microorganisms. Bioengineered cell sensors have been developed to detect specific substances [11,12]. For instance, immobilized algae have been used as optical biosensors to detect environmental pollutants [13]. *Chlorella vulgaris* has also been employed as a biosensor for rapid monitoring of primary-source drinking water [14]. Specially, the light-emission ability of the natural marine bacterium *Vibrio fischeri* (NRRL B-11177) was employed in a sensitive and rapid luminescence-based test, which is described in ISO 11384 [15,16,17].

*V. fischeri* luminesces through the production of luciferase, which catalyzes the oxidation of a long-chain aliphatic aldehyde (RCHO) and a reduced flavin mononucleotide (FMNH_2_) [15]. The free energy in this reaction is released in the form of light at a wavelength of 490 nm (Equation (1)): The presence of acute toxicants that affect this reaction can suppress or extinguish the light emission.

*FMNH*_2_ + *RCHO* + *O*_2_ → *FMN* + *RCOOH* + *H*_2_*O* + *hγ* (490 *nm*) (1)

The presence of acute toxicants that affect this reaction can suppress or extinguish the light emission. Because bioluminescent bacteria are good for real-time monitoring applications, they are widely used for the toxicity assessment of acute toxicants. Usually, cell-based toxicity tests are performed using commercial instruments. However, most of these systems are designed for one-off toxicity assessments, instead of continuous detection. Considering the minimum volume of a bacterial sample (0.2 mL) and the reaction time (30 min) in ISO 11384 [17], a lab-on-a-chip (LOC) device with a stop-flow style will not be suitable for a long time point measurements, let alone continuous monitoring. Currently, bioluminescent cell-based LOC devices are rarely designed for continuous sensing of pollutants [15]. In this study, a continuous-working LOC system was developed to realize the principle of ISO11384 by forming a steady state mixture flow of cells, samples and air bubbles. This system provides a continuous, cost-efficient platform for evaluating the cell toxicity of a broad range of acute chemicals.

## 2. Experimental Section

### 2.1. The Strategy of System Design

The principle of the luminescent bacteria test relies on the inhibition of light emission of *V. fischeri* in response to toxicant effects [17,18]. Generally, an inhibition test is accomplished by tests with specified volumes of the luminescent bacteria suspension in the cuvette. The test criterion is the decrease in luminescence, measured after 15 min and again after 30 min. From parallel measurements of the luminescent intensity changes of standard control samples, the inhibition rate can be determined. Consequently, the level of a toxicant in the sample can be calculated if there is only one toxic chemical in the sample. Here, a continuous-working system based on the same principle as the one-off toxicity assessments in ISO11384 [17] was developed. In this system, the test sample and the luminescent bacteria suspension are continuously introduced into the micromixers at the same flow rate. The mixture then moves through a long spiral micro-channel before reaching the observation chamber for optical measurement. The optical measurement is performed over 20–30 min. When the flow rates and other conditions of the LOC system are fixed, the moving flow forms a stead state for luminescent intensity in the observation chamber. The measured value of steady state can be normalized to the control system on a parallel chip. The inhibition rate based on the light emission can thus be evaluated by comparing the two steady states.

**Figure 1:**
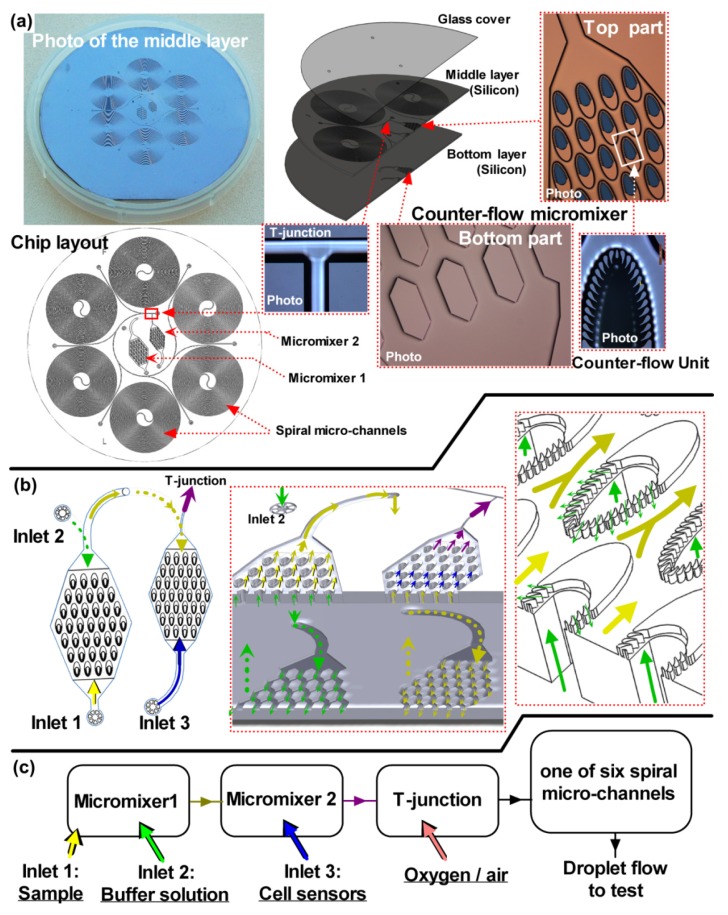
Construction of the cell-based LOC device. (**a**) The chip has three layers, but the three major structures are in the middle layer, including two counter-flow micromixers, a T-junction droplet generator and six spiral micro-channels. A photo of the device is shown in the top left corner. The schematic and micrographs of the counter-flow micromixer are illustrated at the top right part, which houses the counter-flow units (on the middle layer) and the inlet channels (on the bottom layer). The tiny inlet port is located in the center of the counter-flow unit. (**b**) Two counter-flow micromixers are connected in series. The solution flowing in the bottom layer penetrates through the tiny inlet ports in the counter-flow units and then mixes with another solution near the pillar gaps around the tiny inlet port. (**c**) The map of the chip domains is shown at the bottom. The sample, buffer solution and the cell suspension are mixed and used to form droplet within the air flow.

This LOC device is only the core part of the complete continuous-sensing system, which also requires a continuous-sampling module and an external bio-reactor for continuous cultivation of *V. fischeri* cells to continuously provide fresh sensors. Fortunately, these sampling modules, cell separators and bio-reactors have been extensively studied as our research ground [19,20,21,22]. To simplify the experimental system, we used syringe pumps instead of peristaltic pumps to accurately load the test samples. However, a syringe pump would not be suitable for a deployable continuous sampling device. The complex continuous cultivation reactor was not studied in this experimental system to reduce redundant independent factors. Instead, commercially obtained freeze-dried *V. fischeri* cells were used for testing.

### 2.2. Chip Design

The complete continuous-working system is composed of five major components, including a sampling module, peripheral actuating devices (*i.e.*, valves and pumps, *etc*.), a cell-culturing module for *V. fischeri*, a cell-based LOC device and a photomultiplier-tube-(PMT)-based photosensing module. A previously reported non-clogging microconcentrator was employed as the sampling module for drinking water [23]. The external valves, pumps and the *V. fischeri* cells came from commercial sources. The cell-based chip with the optical sensing module was specially designed.

The cell-based chip was composed of three circular layers with diameters of 65 mm (Figure 1). The top layer was a transparent glass cover containing the inlets and outlets. The main structure was integrated within the middle layer. The middle and bottom layers of the chips were composed of 4-inch silicon wafers. There were three domains in the middle layer: the two counter-flow micromixers, the T-junction droplet generator and the six spiral micro-channels with different dimensions.

The counter-flow micromixer used the design of a micro-concentrator to flow two fluids in opposite directions, leading to efficient mixing without a high shearing force [24], which can be destructive to living cells. Although *V. fischeri* cells are robust enough to endure a high shearing force, other cell sensors might be sensitive to this factor (e.g., mammalian cells). The design used for the counter-flow micromixers thus offer a better possibility for expanding the applications of the device.

The three different solutions (*i.e.*, the test sample, the buffer solution and the bacteria suspension) were injected through the three inlets near the micromixers for mixing. A T-junction was designed next to the micromixer domain. At the outlet of the micromixer the solution was encapsulated into droplets within an oxygen or air gas phase flow. The encapsulated solutions were then driven into one of six spiral micro-channels. Only one outlet of the spiral channel domain was open at any given time. The six micro-channels were of the same depth (100 µm), but had different lengths and widths ranging from 100 to 200 µm. They were used to investigate the characteristics of the droplet flow. They also can be used for other applications not shown in this study [25]. The lengths of the spiral micro-channels ranged from 0.83 m to 1.18 m. This facilitated prolonged incubation periods for the cell sensors and toxicant chemicals in the droplets. Thus, the spiral micro-channels were termed the “Time delay channels” (TD-Cs). After incubation in the TD-C, the cells in the droplets were driven into an observation chip with a PMT-based photosensing module. Other potential technical solutions for the photosensing system were studied previously, whereas the PMT-based photosensing module was the most stable one at present. [26,27].

### 2.3. Chip Fabrication and System Installation

**Figure 2:**
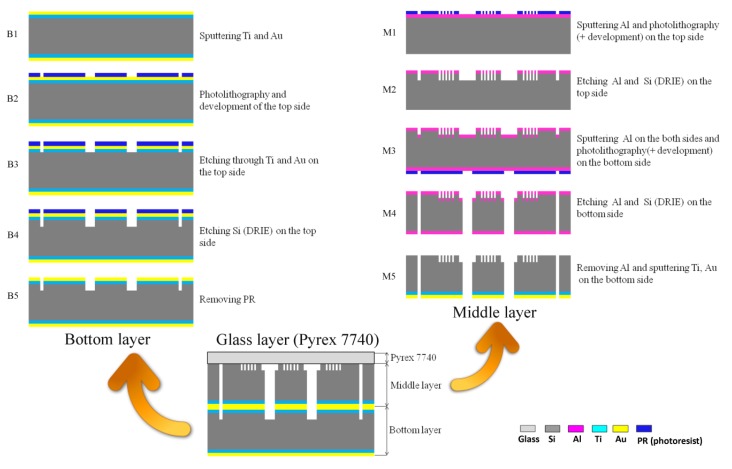
The process flow of the cell-based LOC device. (**a**) The fabrication steps of the bottom layer are marked from Step B1 to Step B5 and diagramed in the top left corner. (**b**) The fabrication processes of the middle layer are illustrated in the top right corner. The steps are labeled from M1 to M5. (**c**) The bonding of the three layers is shown at the bottom.

The middle and bottom layers of the chips were fabricated using a previously-done silicon micromachining process [28]. The middle and bottom layers of the chips were made of 4-inch silicon wafers, for which the processing flow is shown in Figure 2. Silicon-glass anodic bonding was performed to covalently bond a Pyrex 7740 glass wafer on top of the middle layer. The holes for the inlets and outlets on the Pyrex 7740 glass wafer were formed by laser ablation before bonding.

The 5 µL, 10 µL and 20 µL observation chambers were serially fabricated on a polymethylmethacrylate lamina using a HT-4030 laser ablation machine (Feilijia^®^, Liaocheng City, China) [29]. They were then sealed with transparent sealing tape (Biovendis^®^, Mannheim, Germany). A photomultiplier tube (PMT) H10723-01 (Hamamatsu^®^ Photonics, Hamamatsu, Japan) was employed in the detection module for measuring the optical signal from the *V. fischeri* sensors in the observation chamber [18]. The observation chamber was aligned with the center of the effective area of the PMT. Finally, the observation chip with the PMT was wrapped in foil.

**Figure 3:**
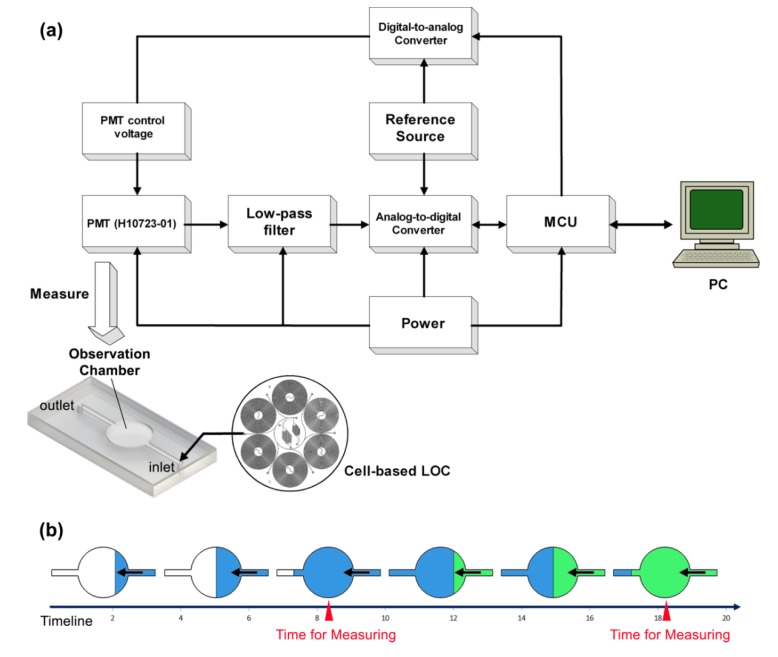
Schematic of the detection system. (**a**) The droplet flow is generated by the cell-based LOC and moved into the observation chamber. The H10723-01 PMT is located on top of the observation chamber to collect the luminescence. The control voltage of the PMT is programed by a DA converter (Analog Devices, Inc.^®^). The voltage output of PMT passes through a low-pass filter to improve the signal-to-noise ratio before AD conversion, the activity of which is controlled by a microcontroller unit (Texas Instruments Inc.^®^, Dallas, TX, USA). The digital signals are transported into a computer (PC) through the USB interface. (**b**) Dynamic diagram of the observation chamber. The droplets flow from the cell-based LOC through the observation chamber continuously. After a short period, the contents of the observation chamber are totally replaced. Accordingly, the detection system measures the observation chamber periodically.

The detection module is shown in Figure 3. A PMT was used as an ultrasensitive detection module with low power consumption and low noise. The optical sensitivity ranged from 230 nm to 870 nm wavelength. The control voltage was regulated by an AD5541 digital-to-analog converter (Analog Devices, Inc.^®^, Norwood, MA, USA). The voltage output of the PMT was transferred to a low-pass filter to upgrade the signal-to-noise ratio before analog-digital conversion by the ADS1258 analog-to-digital converter (Texas Instruments Inc.^®^, Dallas, TX, USA). The ADS1258 was programed using a CC1111 microcontroller unit (Texas Instruments Inc.^®^). Finally, the 24-bit digital signals were imported into a computer (PC) through a USB interface. The application software was developed in Microsoft Visual Studio 2010^®^ [18].

Polytetrafluoroethylene tubes connected the inlets and outlets of the LOC. The outlet of each spiral micro-channel was connected with a HVXD8-5 multichannel valve (Hamilton^®^, Bonaduz, Switzerland). The various water samples, air flows, buffer solutions (1% NaCl and 1% glucose) and 1 × 10^8^ cell/L *V. fischeri* cell solutions (BioToX™ Kit, Aboatox Oy^®^, Masku, Finland) were introduced through digital syringe pumps during testing. The volumetric flow rates were precisely programed.

The volumetric flow rates for the test samples, buffer solutions, bacteria suspensions and air flows were at a constant ratio of 1:1:2:4. The “droplet flow rate” is defined as the total volumetric flow rate of the three liquid solutions. The “response time” of the sensing device is defined as the period from the moment the first droplet is formed to the moment that the observation chamber is filled by the droplets. Because the system continuously monitors the water samples, the observation chamber is measured over a specific period to prolong the working life of the PMT. This period is set to coincide with the period that the droplets in the observation chamber are expelled and replaced by fresh ones. The specific period is defined as the “sampling interval”.

### 2.4. Cell-Based Toxicity Tests in the LOC

The toxicity of the water sample was evaluated through the intensity of emitted light measured by the PMT-based detection module. The luminescence data were calculated using Microsoft Excel^®^ and OriginPro^®^ software. The CuSO_4_ and ZnSO_4_ solutions were serially diluted in deionized water and used as model toxicant samples for testing at concentrations of 0.05 mg/L, 0.5 mg/L, 2.5 mg/L, 12.5 mg/L and 62.5 mg/L. The serial concentrations of both ions ranged from safe levels below the legal limit in potable water to poisonous levels far above the short-term Military Exposure Guidelines (MEG) for water and the Human Lethal Concentration (HLC) [30,31,32]. Deionized water and 2% NaCl were employed as blank solutions. A solution of 60 mg/L cetylpyridinium chloride (Weifa^®^, Oslo, Norway) was selected as a positive control because cetylpyridinium chloride (CPC) is a broad-spectrum bactericidal medicine that can eliminate the bioluminescence efficiently. Fresh solutions of *V. fischeri* were made according to the manual of the BO1243-500 BioToX™ Kit (Aboatox Oy^®^, Masku, Finland). The fresh drinking water sample was taken from the common drinking water pipes in Tønsberg, Norway. The solutions of 50 mg/L potassium dichromate and 4 mg/L 3,5-dichlorophenol were prepared in deionized water and used as artificial toxic water samples in the tests. Their concentrations exceeded not only the legal limit for potable water, but were also larger than the short-term MEG levels for drinking water [30].

After the response time and sampling interval of the LOC system were studied in the initial tests using CuSO_4_ serial solutions, the 20 µL observation chamber was selected as the default chamber volume. Moreover, the droplet flow rate was fixed at 4 × 10^−11^ m^3^/s for all other toxicity tests. The environmental temperature was 295K. The pH, oxygen saturation and ionic strength of the sample were not controlled, as per ISO11384 [17]. However, because this system will be used to monitor drinking water in the future, these parameters should be kept constant; otherwise home users could experience water anomalies without the use of a device. The relative luminescence unit (RLU) was measured three times for each sample. The bioluminescence data for ZnSO_4_ were analyzed by linear and non-linear fitting methods using OriginPro^®^ software.

## 3. Results and Discussion

### 3.1. The Cell Toxicity Tests on the Chip

After our initial experiments, the operating parameters of the system were standardized. The observation-chamber (volume of 20 µL) and the spiral micro-channel (200 µm in width, 0.83 m in length) were employed as defaults. 

**Figure 4:**
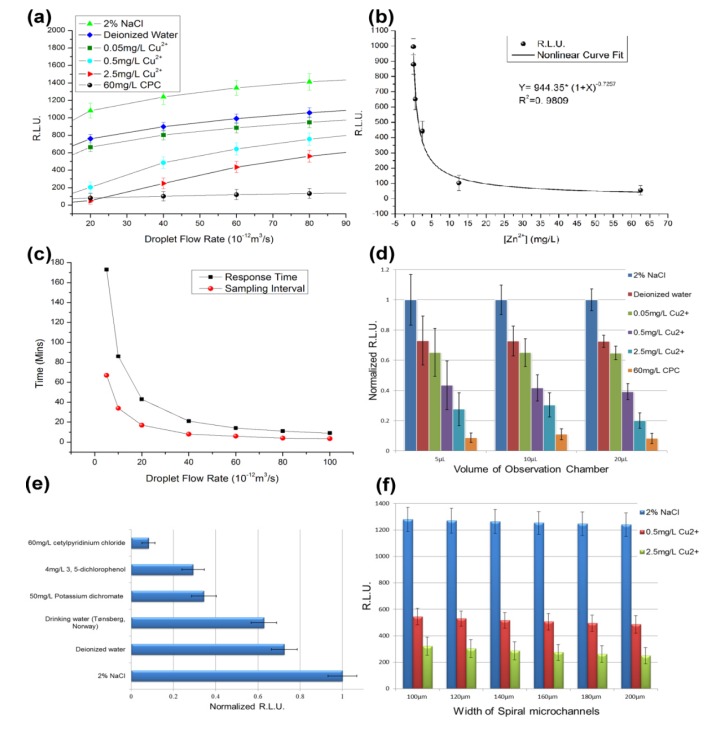
Toxicity test results for the *V. fischeri* cell-based sensing system. (**a**) The six sample solutions (2% NaCl, deionized water, 0.05 mg/L Cu^2+^ solution, 0.5 mg/L Cu^2+^ solution, 2.5 mg/L Cu^2+^ solution and 60 mg/L cetylpyridinium chloride (CPC)) were tested at several droplet flow rates [18]. (**b**) Nonlinear regression between [Zn^2+^] and the RLU data. (**c**) Response time and sampling interval of the sensing device. (**d**) Observation chambers with volumes of 5 µL, 10 µL and 20 µL were tested. (**e**) The drinking water sample and five artificial solutions were tested in the cell-based system, including 2% NaCl, deionized water, a drinking water sample taken from Tønsberg, Norway, 50 mg/L potassium dichromate, 4 mg/L 3,5-dichlorophenol and 60 mg/L CPC. (**f**) Parallel tests for the six spiral microchannels.

The six Cu^2+^ solutions were tested in the cell-based sensing device for several droplet flow rates (Figure 4a). With the same operating parameters, the RLU in the group with 2% NaCl were the highest because the 2% NaCl solution is close to the isosmotic solution of *V. fischeri*. The RLU for the deionized water group was approximately 25% lower than the 2% NaCl group. The group that used the 0.05 mg/L Cu^2+^ solution showed very similar results to the deionized water group (Figure 4a). However, the solutions of 0.5 mg/L Cu^2+^ and 2.5 mg/L Cu^2+^ showed some toxicological effects on the *V. fischeri* cells (Figure 4a). When the droplet flow rate was 4 × 10^−11^ m^3^/s, the RLU was less than 50% that of the isosmotic group (Figure 4a). The results for the positive control group (60 mg/L CPC) were in line with expectations (*i.e.*, their bioluminescent signals were close to zero) (Figure 4a).

When the droplet flow rate decreased below 2 × 10^−11^ m^3^/s, the response time increased to more than 30 min (Figure 4c). For the safety of home users, 4 × 10^−11^ m^3^/s was selected as the standard droplet flow rate to limit the response time to within 30 min. When different observation chambers and spiral microchannels were tested in the parallel experiments, the results showed that using a smaller observation chamber led to larger relative errors (Figure 4d), whereas the switching among different spiral microchannels had no significant impact on the results (Figure 4f). Thus, the observation-chamber of 20 µL in volume was preferred.

The standard toxicity assessment using *V. fischeri* cells as biosensors is based on inhibition of luminescence. The toxicity is usually represented as the EC_50_, which is the effective concentration of the tested chemical at which 50% of the luminescence is inhibited [33]. The reported EC_50_ values for copper (II) exposure of *V. fischeri* range from 0.15–0.58 mg/L at an exposure time of 30 min [34]. In this study, the *V. fischeri* cells and water sample were mixed in the second micro-mixer. After that the cell passed through a long TD-C channel and moved into the observation chamber for measurement. Hence, the total volume of the TD-C channel and the observation chamber divided by the droplet flow rate give the exposure time for each *V. fischeri* cell (Figure 3). As the volume of the observation chamber is larger than the other parts of the chip in total, the exposure time approximates the response time (Figure 4c). When 4 × 10^−11^ m^3^/s is selected as the droplet flow rate, the exposure time is approximately 30 min. Consequently, the EC_50_ value for copper (II) is approximately 0.5 mg/L in Figure 4a because the RLU of the 0.5 mg/L Cu^2+^ group is approximately 50% that of the deionized water group at a droplet flow rate of 4 × 10^−11^ m^3^/s. The EC_50_ values for the toxicological tests in the microfluidic device were in line with the values for traditional *V. fischeri* tests, but the standard reference values for the on-chip *V. fischeri* tests ought be modified due to the different physical environment in the microfluidic chip.

The Zn^2+^ solutions were also measured using the standard settings and the results were analyzed by nonlinear regression (Figure 4b). The nonlinear curve for the Zn^2+^ concentration and the respective RLU were obtained with a correlation coefficient of 0.9809. Based on the nonlinear regression equation, the calculated EC_50_ value for zinc (II) was 1.40 mg/L, while the reference EC_50_ value in ISO11348 was 2.17 mg/L [17,35]. The luminescence-based toxicity tests for copper (II) and zinc (II) indicated that the on-chip *V. fischeri* tests provide comparable results to the traditional tests. When the concentration of Zn^2+^ was below 5 mg/L, the RLU of observation chamber decreased sharply as the concentration of Zn^2+^ increased. However, when the concentration of Zn^2+^ was above 10 mg/L, the RLU changed only slightly. This indicates that the cell-based LOC device can be used to quantify zinc ions when the Zn^2+^ concentration has the same order of magnitude as the standard for Zn^2+^ in drinking water (1 mg/L). For the 0.05 mg/L, 0.5 mg/L, 2.5 mg/L Zn^2+^ solutions and deionized water (0 mg/L), only a poor linear relation was obtained by linear regression with a correlation coefficient less than 0.8 (data not shown). Thus, this sensing system is useful as a qualitative tool, rather than as a quantitative test, even if there is only one chemical species in the sample. Similar results were obtained for the solutions of CuSO_4_.

The sample of real drinking water and the two artificial samples of polluted water were tested in the cell-based system. The samples were significantly different (Figure 4e). The RLU data for the real drinking water sample was close to that of the deionized water group (the blank control), whereas the solutions of 50 mg/L potassium dichromate and 4 mg/L 3,5-dichlorophenol showed half of the RLU value of the deionized water group. According to the test criterion for the device, for a larger concentration of an acute toxicant, the test will produce a smaller RLU Obviously, the polluted water and qualified water can be easily distinguished by the cell-based LOC. However, the water samples were difficult to distinguish by the senses of the volunteers.

The reduction in the RLU for the deionized water reflects the impact of inappropriate salinity on the cell sensors, but this influence was limited. In addition, copper and zinc in the water are not toxic to humans if their concentrations are below the upper limits for the World Health Organization standards for drinking water quality (2 mg/L) [32,35]. It is thus reasonable that the response of the cell sensors in the tests of 0.05 mg/L Cu^2+^ or Zn^2+^ solutions are similar to the responses for deionized water.

The system is more appropriate as an early warning device to assess possible threats of chemical toxicants in water. It is worthwhile to note that the cell-based LOC device was designed to assess the toxicity of chemicals in water, not their concentrations. The measurement of toxicity is not equivalent to the chemical concentration. As a qualitative monitor for screening toxicants, it is not necessary to distinguish the details of toxicants. The major advantage of the sensing system lies in its ability to continuously monitor the water. Samples and cell sensors should also be imported into the chip so that it can run continuously and smoothly. When all the parameters are fixed, the changes in the steady state in the observation chamber can be attributed to changes in the chemical compositions of the samples. On the other hand, the system might generate false signals because there are many variables that influence the operation of the system, such as environmental temperature, oxygen concentration in the air and the presence of other inorganic or organic compounds in the water. Therefore, the device has to work in a stable environment and might be difficult to deploy in the field.

### 3.2. The Design of the Detection Module

Before the installation of the PMT system, we attempted to observe the luminescence signal from the cell-based chip in a dark room using a microscope system with a charge coupled device (CCD) camera. However, no visible image could be obtained even when using the ultimate sensitivity of the CCD detector because the bioluminescence of several hundred *V. fischeri* cells in one droplet is still too weak to serve as a light source inside the microscope. This trial indicated that the conventional CCD detector in a common microscope system is not sufficient for measuring the weak bioluminescent signals in the droplets. Another unsuccessful trial using the PMT to measure the droplet flow was tested directly on the microfluidic chip. The effective area in the PMT was placed towards the outlet of the spiral microchannel, but the signals were too weak and not stable. This may have resulted from the low energy density of the living organisms and their inferior luminous efficiency. Therefore, the observation chambers were employed to increase the luminescence level for each measurement.

If the *V. fischeri* cells on the chip are analogous to fish, a spiral microchannel is analogous to a long river, while an observation chamber represents a huge lake. The cell sensors in the spiral microchannels and observation chambers can in theory reflect the quality of the water samples, but each observation method is different. In this study, the average state of the cell sensors in the “lake” is measured to judge the water quality. In fact, the volume of the observation chamber is a critical factor in the detection module because the volume and the droplet flow rate can determine the total reaction time for the *V. fischeri* cells in the toxicant sample and thus dominate the residual activity of the cells and the measured RLU. When the cell sensors are measured by counting the frequency of bioluminescent droplets at the end of TD-Cs, the switch of the TD-Cs might result in a significant difference.

### 3.3. Distinguishing Features and Ethical Issues of the Household LOC System

Compared with existing assays and platforms for monitoring drinking water quality, the bioluminescent-cell-based LOC device was developed for home users rather than for the water industry or aquatic environment researchers [2,36,37]. The principle of ISO11382 was realized on the miniaturized LOC device, which drastically reduced the size and maintenance cost. The bioluminescent cells make the microfluidic chip easy to use because no light source is required. The LOC system was also designed to work continuously for a long time. Given sufficient fresh culture medium and energy, this system can theoretically run indefinitely. It is obvious that the responses to broad ranges of toxicants and the narrow measured range of living cells provide a comparative early warning system for home users because they only need qualitative results.

The safety of the cell-based LOC device has to be considered when used at home. First, bacteria are employed inside the LOC system; thus, home users should avoid touching the bacteria [38]. In case of possible leakage, the system is suggested to be installed inside toilets, where the waste or leakage liquid can be directly discharged through the sewer system. *V. fischeri* can also be inactivated with quicklime powder. An optional method for waste treatment is to collect the waste liquid in a container of quicklime powder. Given the operating parameters, the estimated annual consumption of quicklime will be 0.3 Kg. The powder can be renewed during the annual inspection.

## 4. Conclusions

A *V. fischeri* cell-based microfluidic device was developed and validated as a promising continuous-working analyzer against acute toxicants in drinking water. The system uses living organisms and thus has an inherent advantage at discriminating toxic substances, although its sensitivity may not be outstanding. Not all of the intended functions of this device have been realized, but the sensing system achieves the main goal of assessing the acute toxicity of chemicals in drinking water, instead of quantifying the concentrations of chemicals. Furthermore, the broad-spectrum analyzer is capable of continuous sensing of unexpected or unknown systemic acute toxicants in drinking water as an early warning monitor, which meets the demands of home users.

The analytical method developed in this study is based on the measurement of bioluminescent intensity in a large observation chamber (20 µL). The method is conservative and robust. Its response time is approximately 20 min, similar to the total reaction time of ISO 11348. However, the cell-based system should be further developed into a complete digital microfluidic platform with a nearly real-time response. In this case, the PMT-based detection module should be improved and directly integrated in the outlet of the TD-Cs to build a total analytical system. Ongoing work also involves the development of a telecommunication module, an eco-friendly waste-treatment module and an external or integrated micro-chemostat that can continuously introduce *V. fischeri* cells. In the future, this household cell-based sensing system could persistently monitor the quality of drinking water at a low cost. Once suspicious signals appear, both the inhabitants and drinking water suppliers would receive a prompt alarm to avoid possible accidents.
